# Perspectives of African American Church Leaders in Response to COVID-19 Emergency Preparedness and Risk Communication Efforts Within a Community Engaged Research Partnership: COVID-19 emergency risk communication

**DOI:** 10.1017/dmp.2023.182

**Published:** 2023-10-13

**Authors:** Mathias Lalika, Manisha Salinas, Gladys B. Asiedu, Clarence Jones, Monisha Richard, Jamia Erickson, Jennifer Weis, Adeline Abbenyi, Tabetha A. Brockman, Irene G. Sia, Mark L. Wieland, Richard O. White, Chyke A. Doubeni, LaPrincess C. Brewer

**Affiliations:** 1 Division of Preventive Cardiology, Department of Cardiovascular Medicine, Mayo Clinic, Rochester, MN, USA; 2 Center for Health Equity and Community Engagement Research, Mayo Clinic, Jacksonville, FL, USA; 3 Robert D. and Patricia E. Kern Center for the Science of Healthcare Delivery, Mayo Clinic, Rochester, MN, USA; 4 Hue-Man Partnership, Minneapolis, MN, USA; 5 Volunteers of America, Incorporated, Minneapolis, MN, USA; 6 Thrivent Financial, Incorporated, Rochester, MN, USA; 7 Center for Clinical and Translational Science, Mayo Clinic, Rochester, MN, USA; 8 Center for Health Equity and Community Engagement Research, Mayo Clinic, Rochester, MN, USA; 9 Division of Infectious Diseases, Mayo Clinic Department of Medicine, Rochester, MN, USA; 10 Division of Community Internal Medicine, Mayo Clinic Department of Medicine, Rochester, MN, USA; 11 Division of Community Internal Medicine, Mayo Clinic Department of Medicine, Jacksonville, FL, USA; 12 Department of Family and Community Medicine, The Ohio State University Wexner Medical Center, Columbus, OH, USA

**Keywords:** Covid-19, Emergency Preparedness, risk communication, community-based participatory research, community-academic partnerships

## African-American Churches are Key Partners in Addressing Health Challenges

The COVID-19 pandemic disproportionately affected communities of color, particularly African-Americans (AAs), exacerbating health disparities.^1^ Since 78% of AAs report belonging to religious organizations, the Fostering African-American Improvement in Total Health! (FAITH!) Program leveraged a successful, longstanding community-academic partnership in April 2020,^2–6^ by providing emergency preparedness (EP) plans and COVID-19 information to AA churches in Rochester and Minneapolis-St. Paul (MSP), MN.^7^ This mixed-methods study evaluated the initiative’s impact on EP promotion within AA communities during the pandemic; assessing church readiness for future public health emergencies, intervention engagement, dissemination, and satisfaction.

## Methods

### Intervention/Context

From April to May 2020, an 8-week EP risk communication intervention was co-developed with AA churches in Rochester and MSP (AAs 9.1% and 18.4% of population, respectively). In the intervention areas, an estimated 60% of AAs attend Christian congregations. Employing a community-based participatory research approach and a framework adapted from the Centers for Disease Control and Prevention (CDC) Crisis and Emergency Risk Communication framework, a needs assessment was conducted to understand pandemic-related challenges faced by the AA community. Intervention details have been previously published.^7^ Based on the needs assessment, a faith-based, COVID-19 EP manual was electronically communicated.^7^ The manual offered step-by-step guidance for structuring church EP initiatives and establishing EP teams (EPTs). Additionally, a culturally tailored social marketing campaign was launched to communicate inspirational, health, and financial, as well as social support messages. The “FAITH! & COVID-19 Spread the Word!” newsletter, co-developed with community leaders, was sent weekly to congregations via email containing scripture-based information to reinforce messages.

### Data Collection

The Mayo Clinic Institutional Review Board approved this study. Following informed consent, AA church leaders participated in individual, 1-hour, semi-structured interviews (June–July 2020) via videoconferencing and telephone using a standardized moderator guide. All interviews were recorded and transcribed. Participants received US $50 cash card for interview completion. Churches also received US $500 monetary incentives to enhance EPTs.

### Outcomes

We assessed whether churches had an established EPT and the perceived level of church EP at pre- and post-intervention. Furthermore, the intervention was evaluated on engagement, dissemination, satisfaction, and recommendations/suggestions for sustainability.

### Analyses

Participant characteristics were summarized using mean, median, and proportions. Initial themes and categories were compiled using an integrated approach of inductive codes emerging from the data and *a priori* codes derived from interview questions. Multiple readings were conducted to identify preliminary themes. Quantitative analysis utilized SPSS Statistics Version 25 (IBM Corp., Armonk, NY, USA) and qualitative analysis used QSR NVivo software, v10 (Doncaster, Victoria, Australia).

## Results

### Quantitative Results

Fifteen church leaders were interviewed (response rate: 47% [15 / 32 churches]). Mean age was 60 (SD 9.3) years, with a majority being women (n = 12, 80%). Most were pastors and health coordinators (n = 8, 54%). Representing churches were mainly in MSP (n = 12, 80%); and the median congregation size was 85 members.

Table 1 displays church EP and intervention evaluation. Pre-intervention, 73% (n = 11) of the churches had a health ministry, while only 27% (n = 4) had an established EPT. About 40% (n = 4) of church leaders felt their churches were prepared for a health crisis. Post-intervention, 73% (n = 11) had established an EPT (or equivalent structure), and 3 leaders planned future EPTs. All 15 churches reported increased readiness for the ongoing pandemic and future health crises.

The FAITH! EP intervention (n = 8, 53%) ranked among the top 3 reliable COVID-19 information sources, alongside the governor’s briefings (n = 8, 53%) and the CDC (n = 7, 47%). Of 13 leaders receiving weekly emails, 85% (n = 11) shared information with their congregations. Among 7 churches receiving the EP manual, 57% (n = 4) used it to establish or enhance EPTs. Although 67% (n = 10) knew of the FAITH! Facebook page, only 30% (n = 3) interacted with its content. All leaders who received emails and the EP manual found them helpful for mitigating the pandemic.

### Qualitative Results

Thematic analysis of semi-structured interviews yielded 4 themes (Table 2) with no differences by interview mode (videoconferencing vs. telephone).

“Perceived Level of Church EP” indicated pre-intervention unpreparedness for health crises like the pandemic. Improved EP post-intervention was attributed to adherence to recommended standards, enhanced knowledge, technology integration, and better communication strategies.

“Intervention Engagement/ Dissemination” underscored the EP manual’s adaptability and its role in launching EPTs. The majority of church leaders valued the weekly emails, finding them highly informative and relevant in addressing pandemic-related needs. Instead of forwarding the emails, leaders often summarized key points for distribution through church communication channels (e.g., email and websites).

“Intervention Satisfaction” revealed that the FAITH! EP intervention significantly impacted EP via the manual. The manual was praised for its accessibility and cultural appropriateness, although some participants cited time constraints and pandemic-related demands as hindrances to fully reviewing and implementing its contents.

The “Intervention Sustainability” theme featured church leaders’ recommendations for long-term impact. They proposed sustaining the intervention by including community health needs (e.g., cancer screening), expanding prevention efforts (e.g., distributing hand sanitizers), incorporating non-COVID-19 healthy lifestyle information, and converting the manual into webinars for increased accessibility.

## Discussion

This community-centric intervention improved EP in AA churches by establishing new, and enhancing existing EPTs for health crises, including infectious disease outbreaks. The COVID-19 EP manual aided crisis mitigation efforts led by the EPTs. Participants found the intervention messaging reliable and useful. Similar initiatives addressed vulnerable communities’ needs elsewhere in the US but lacked an EP focus.^8,9^ This study underscores the need for immediately deployable, sustainable EP tools (e.g., the FAITH! EP intervention) for church leaders to effectively navigate disasters and keep parishioners safe.

The small sample size and localized geographic region limits generalizability of findings beyond these communities. Nevertheless, we achieved interview saturation, underscoring the validity of the findings and offering valuable insights for enhancing EP in under-resourced AA communities. Effective information communication is crucial in the digital transformation era. Participants recommended concise formats, leading to a FAITH! program webinar series covering EP essentials. This highlighted the need for user-friendly digital toolkits in future interventions. Importantly, contextualizing EP strategies is crucial, particularly in socioeconomically disadvantaged communities where unmet or neglected needs are prevalent.^10^

### Conclusion

Equipping churches equitably with EP resources and tailoring resources through EP assessment are pivotal for effective crisis response in AA communities.

## Figures and Tables

**Table 1.** Church emergency preparedness and evaluation of the FAITH!emergency preparedness and risk communication initiative

	N = 15 N (%)
**Pre-intervention**	
Age, years, mean (SD)	60 (9.3)
Sex	
Female	12 (80)
Male	3 (20)
Leadership role	
Pastor	4 (27)
Health coordinator	4 (27)
Trustee	2 (13)
Administrator	2 (13)
Bishop	1 (7)
Pastor’s spouse	1 (7)
Outreach leader	1 (7)
Church location	
Minneapolis-St. Paul Area	12 (80)
Rochester	3 (20)
Church congregation size, median (IQR)	85 (60, 313)
Established emergency preparedness team	4 (27)
Preparedness level for a health crisis	
Prepared	6 (40)
Not prepared	9 (60)
**Post-intervention**	
Established emergency preparedness team	11 (73)
Preparedness level for a health crisis	
Prepared	15 (100)
Impact of the intervention on emergency preparedness (n = 14)	
Perceived impact	11 (73)
No Perceived impact	3 (20)
Useful, reliable COVID-19 information sources	
FAITH! program	8 (53)
Minnesota Governor’s briefings	8 (53)
Centers for Disease Control and Prevention (CDC)	7 (47)
Minnesota Department of Health	6 (40)
Faith-based organizations	5 (33)
News Channels	4 (27)
Engagement and dissemination of weekly emails	
Received	13 (87)
Disseminated (n = 13)	11 (85)
Satisfaction with weekly emails (n = 13)	
Helpful	13 (100)
Engagement and dissemination of the manual	
Received	7 (47)
Utilized (n = 7)	4 (57)
Satisfaction with the manual (n = 4)	
Helpful	4 (100)
Awareness of Facebook messaging	
Aware of FAITH! Facebook page	10 (67)
Familiarity with COVID-19 information on FAITH! Facebook Page [n = 10]	
Familiar	5 (50)
Not familiar	5 (50)
Engagement and dissemination of the Facebook page	
Followed the page	6 (60)
Liked, shared, or promoted individual posts	3 (30)
Satisfaction with the Facebook page (n = 10]	
Helpful	4 (40)
Not at all helpful	1 (10)
Do not know	5 (50)

**Table 2.** Perceptions of FAITH! emergency preparedness and risk communication initiative

Major Themes	Illustrative quotes

Perceived level of emergency preparedness for a health crisis before and after the intervention	*“I’d say adequately. I think we’ve, you know, we’ve kind of found a system that fits our bandwidth for where we are right now.”*
	*“… we established different safety protocols for the food shelf.”*
	*“We had to work with other organizations, bigger food shelves, and the state department, just to make sure we were complying and doing things in a way that was safer.”* [Female, 48 years]

Intervention engagement and dissemination	*“Very helpful in setting up our own team and coming up with guidelines for reopening. Especially precautionary guidelines. Now of course, the purpose of the resource is for us to set up our own team, which we did, when the church reopened. It was a good resource for us.”* [Male, 53 years]
	*“How to organize and set up the [emergency preparedness] plan and how to safely implement the plan: that information came straight from the faith-based details they were sending out.”* [Female, 53]
	*“Based on the written guidelines, someone who didn’t have any administration background could use the tool that they had in place.”*
	*‘It was very, very helpful. Because … it was information. We didn’t have to be looking around, sourcing for this information.”* [Female, 53]

Intervention satisfaction	*“They [weekly emails/ messaging] are, in terms of content and medical information, both spiritual and informative. Some resources are also economical. Hence, it has balanced content.”* [Male, 53]
	*“[The information is] coming from a personal perspective; it’s coming from a project that we’re participating in so there’s a relationship. All of it is helpful. We trust [the investigator], and we’re trusting Mayo.”* [Female, 73]
	*“I would say because the information is coming from Mayo and most people know the reputation of Mayo, I think that people are more inclined to engage in the links more than if it was coming from somewhere else. Knowing the work that Mayo is doing and has done, and its’ reputation, I think people will engage in it more readily.”* [Female, 60]
	*“It’s always helpful for me, and the information is always enlightening. If people needed resources there are so many updates, and encouragement for people to share their own resources. Hence, for some of the updates, even if I don’t listen to the Governor, I see the [FAITH! Program Facebook] page and I’m aware of what decision was made from [that] page.”* [Female, 62]

Recommendations and suggestions for intervention sustainability	

	**Integrate non-COVID-19 healthy lifestyle information**
	*“For this particular program, for FAITH! at first, it was healthy heart care and cholesterol and that kind of stuff. That’s because the program appealed to us, because we were just starting a fitness program and it would go hand-in-hand with being mindful of better eating and workout habits.”* [Female, 48]
	*“Resources for the church, and information/ news we don’t know, for example, if they’re doing antibody testing or something that is not as well known, innovations from other churches, etc. should be included. Something that would give us ideas on how better to connect during this time when it’s harder to connect. If the newsletters could have more value, and go a little deeper in the information presented (e.g., safe activities to do outside in nicer weather while maintaining social distancing such as tennis, biking, etc.). I would definitely share such helpful tips more.”* [Female, 48]
	**Increase overall prevention efforts**
	*“I would add that one of the things that I thought before the pandemic and one of the absolute needs in the African American community, specifically, is how to live a healthy lifestyle. Healthy eating, access to healthcare, dental care, etc. You know, people don’t really understand how your teeth are tied to your health. Information such as having mammograms, cancer screenings, etc., should also be incorporated. Taking some of the stigma and fear from some of the tests, I think would be very, very helpful because some folks don’t go to the doctor because they are scared.”* [Male, 58]
	**Incorporate health needs prevalent in the community**
	*“We all pray COVID will pass, but diabetes, lupus, and heart disease, amongst others are still going to be here.”* [Male, 58]
	**Ensure messages reach appropriate audiences**
	*“I don’t know if there was another way other than Facebook; a lot of our seniors don’t use Facebook and would probably would benefit a lot more than our younger members. I know that we’re in this age of technology and it’s wonderful, but it also leaves out some individuals that also need that information. They’re not in tune with new-age learning technology.”* [Female, 60]
	*“It would be great if someone could actually hold a webinar [on emergency preparedness manual]. So you don’t have to be reading all the lines, and can just at your convenience, just view instead of reading.”* [Male, 53]

## Abbreviations.

AA: African American
CDC: Centers for Disease Control and Prevention
EP: Emergency Preparedness
EPT: Emergency Preparedness Team
FAITH!: Fostering African-American Improvement in Total Health!
MSP: Minneapolis-St. Paul
